# Digital Gait Biomarkers for Parkinson’s Disease: Subject-Wise Validated Explainable AI Framework Using Vertical Ground Reaction Force Signals

**DOI:** 10.3390/bioengineering13030360

**Published:** 2026-03-19

**Authors:** Moonhyeok Choi, Jaehyun Jo, Jinhyoung Jeong

**Affiliations:** 1 Department of Electronic and Communication Engineering, Catholic Kwandong University, 24 Beomil-ro 579 Beongil, Gangneung-si 25601, Republic of Korea; ansgur110@cku.ac.kr; 2 Department of Digital Healthcare, Catholic Kwandong University, 24 Beomil-ro 579 Beongil, Gangneung-si 25601, Republic of Korea; jh_507@cku.ac.kr; 3 Department of Healthcare Management, Catholic Kwandong University, 24 Beomil-ro 579 Beongil, Gangneung-si 25601, Republic of Korea

**Keywords:** Parkinson’s disease, vertical ground reaction force, explainable artificial intelligence, severity estimation

## Abstract

Parkinson’s disease (PD) is associated with progressive gait deterioration; however, widely used clinical scales such as the Hoehn & Yahr (H&Y) stage are limited in capturing continuous severity changes due to subjectivity and discrete grading. This study proposes a two-stage explainable AI framework using vertical ground reaction force (VGRF) signals to achieve reproducible PD detection and continuous severity estimation. In the first stage, three deep learning models, temporal convolutional network (TCN), BiGRU with attention, and FCNN-Transformer, were trained using windowed VGRF signals under repeated subject-wise data segmentation. All models achieved high discrimination performance (AUC ≥ 0.93), with FCNN-Transformer showing the highest mean AUC (0.940) and statistically superior performance (paired Wilcoxon test, *p* < 0.05). Stability-based explainable AI using Integrated Gradients consistently identified variability-related VGRF features as the most informative, which were also significantly different between groups at the data level (*p* < 0.001, FDR-corrected). In the second stage, XGBoost regression was applied to PD subjects to predict continuous H&Y severity, achieving strong correlation with clinical grades (Spearman ρ = 0.921, *p* < 0.001), low error (MAE = 0.158, RMSE = 0.241), and high determination (*R*^2^ = 0.953). This shows that gait-based features are a sensitive enough signal to continuously quantify disease progression. In addition, in the TREND prospective longitudinal cohort (*n* = 696), wearable walking indicators differed significantly from those of non-patients prior to diagnosis, and a decline in walking pace was observed approximately four years before Parkinson’s disease diagnosis, providing the basis for early screening and monitoring using gait-based digital biomarkers. These results demonstrate that gait-based digital biomarkers can objectively quantify both PD presence and disease progression. The proposed framework provides a reproducible, explainable, and clinically interpretable AI-based decision support approach for PD assessment.

## 1. Introduction

Recent advancements in artificial intelligence (AI) technology and wearable sensor-based biosignal analysis technology have greatly expanded the possibility of using digital biomarkers in the area of neurological disease evaluation [1]. In particular, due to its non-invasive and repeatable nature, gait-based biosignals have attracted attention as a key digital phenotype for early diagnosis of neurodegenerative diseases, monitoring disease progression, and evaluating treatment response [2]. However, despite these technological advancements, there are still structural limitations in terms of reproducibility, generalizability, and interpretability in the construction of clinically applicable AI-based gait analysis systems.

Parkinson’s disease is a typical progressive neurodegenerative disease characterized by motor dysfunction such as dysphonia, muscular stiffness, progression, and postural instability, and progressive changes in walking patterns appear with the progression of the disease [3]. These changes are directly linked to decreased mobility, increased risk of falls, and decreased quality of life, and walking disorders are considered a key clinical symptom that significantly affects the daily function performance and independence of patients with Parkinson’s disease [4]. In clinical settings, clinical evaluation tools such as neurological examination and the Hoehn & Yahr (H&Y) scale are mainly used for the diagnosis and staging of Parkinson’s disease, but these scales imply structural constraints such as subjectivity of evaluators, issues of agreement between evaluators, variability according to patient’s cooperation, and limitations of reflecting continuous severity due to staged structures [5]. These limitations further highlight the need for objective and quantitative adjuvant indicators [6].

Against this background, there is a growing interest in physiological signal-based digital biomarkers, and in particular, gait analysis has attracted attention as a promising approach to non-invasive assessment of motor symptoms in Parkinson’s disease. Among them, VGRF signaling directly reflects the interaction between the foot and the ground while walking and has the advantage of being able to quantitatively assess neuromuscular control and motor stability through force distribution and variability during the walking cycle [7]. Due to these characteristics, VGRF has been proposed as a potential digital biomarker capable of sensitively capturing abnormal walking patterns in patients with Parkinson’s disease. Random or record-wise segmentation that ignores subject boundaries in deep learning studies using biological signals causes subject-level data leakage, which is a major reason for consistently overestimating the model’s predictive performance. Several recent literature studies also warn that such data leakage leads to severe performance degradation in external clinical cohorts and is one of the most persistent limitations that hinder clinical readability and cross-cohort generalizability of artificial intelligence models [8,9].

In previous studies, VGRF-based features are useful in distinguishing Parkinson’s disease patients from normal people, and some features have also been reported to be associated with disease severity [5]. However, a number of prior studies relied on manual-based feature design or traditional machine learning models, and by using sample- or window-level data segmentation strategies, the subject-level data leakage problem was included in the learning and evaluation stage of the same subject’s data [10]. These structural problems pose the risk of overestimating the generalization performance in actual clinical settings and can seriously impair the reproducibility and clinical reliability of research results.

Recent advancements in deep learning techniques suggest the possibility of automatically learning meaningful expressions from gait signals and report improved classification performance compared with existing methods [6]. However, deep learning-based approaches also have two key limitations. First, the subject-wise iterative verification strategy has not been sufficiently applied in many studies, so the stability and reproducibility of the model performance have not been systematically verified [11]. Second, the black box characteristics of deep learning models limit clinical interpretability, and despite the introduction of XAI techniques such as SHAP and Integrated Gradients, most of them are limited to single-model- or single-segmentation-based explanations, so verification of the stability and repeatability of explanation results is insufficient [12].

In addition, it is reported that the input expression method and model structure selection have a significant impact on predictive performance and interpretability in time series biosignal analysis such as gait signals [13]. There is a limit to capturing these characteristics sufficiently with only a single model structure because the VGRF signal contains both local pattern and temporal dependence within the walking cycle. As a result, this study is designed to enable performance evaluation and explainability analysis that are not biased toward a specific model structure by utilizing multiple deep learning backbone structures based on different time series modeling assumptions in parallel [6].

Furthermore, existing studies have mainly focused on the binary classification problem that distinguishes Parkinson’s disease patients from normal people, and the problem of quantitative prediction of clinically important disease severity has not been relatively sufficiently addressed [14]. However, in the actual clinical environment, continuous assessment of disease progression as well as the presence or absence of disease is equally important in terms of treatment strategy determination, prognosis prediction, and disease progression monitoring. According to recent clinical studies, continuous monitoring in daily life using wearable sensors is reported to play a key role in objectively capturing the variability of patients’ symptoms, allowing neurologists to optimize drug treatment doses and lower hospitalization rates. In addition, by providing quantitative data on motor symptoms for a long time beyond the limits of one-off clinical evaluation, personalized treatment interventions and information-based clinical decision-making are possible [15,16]. In particular, sufficient evidence has not yet been accumulated as to whether walking-based features can reflect not only the presence of the disease but also the degree of disease progression. Most of the existing artificial intelligence-based Parkinson’s disease gait analysis studies have been conducted only through binary classification tasks that distinguish between patients and normal groups. Although artificial intelligence has achieved high technological performance in the diagnostic field, research on continuous and quantitative estimation of disease severity, such as the Hoehn and Yahr (H&Y) scale or UPDRS, with only sensor data remains a challenging task [6].

To overcome these limitations, this study proposes a two-stage explainable artificial intelligence framework centered on subject-wise verification based on a windowed VGRF signal [17]. In the first stage, the generalizability and stability of the model performance are systematically evaluated by performing Parkinson’s disease binary classification under repetitive subject-wise data segmentation using a number of deep learning backbone models [18]. In the second stage, the Hoehn & Yahr severity is continuously predicted through a regression model only for subjects classified as Parkinson’s disease, and it is analyzed whether the walking signal can quantitatively reflect not only the disease presence but also the disease progression [19].

Specifically, this study aims to overcome the limitations of explanations relying on a single model or a single experiment and derive reproducible and reliable walking biomarker candidates by introducing a stability-based explainable artificial intelligence (XAI) strategy that selects only features that appear consistently across iterative segmentation and different model structures [20]. The derived candidate features were further verified through nonparametric statistical tests, effect size analysis, and FDR correction to simultaneously secure explainability, statistical validity, and clinical interpretability [21].

This study differs from previous studies in that it presents a digital biomarker-based clinical decision-making support structure that simultaneously secures reproducibility, reliability, and clinical interpretability in Parkinson’s disease gait analysis studies through a framework that integrates subject-wise iterative verification, stability-based XAI, and continuous severity regression [22].

## 2. Materials and Methods

**Figure 1:**
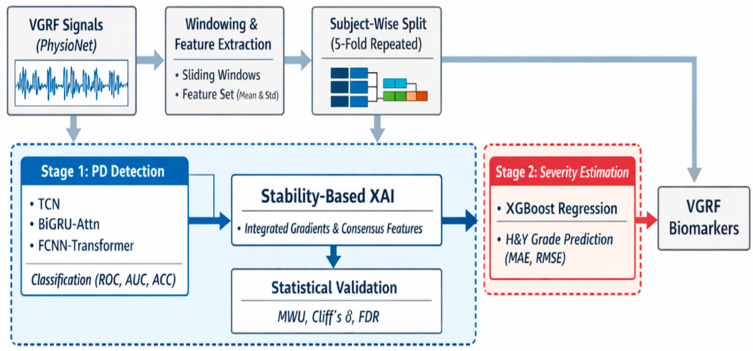
Overview of the two-stage explainable artificial intelligence framework of this study for Parkinson’s disease assessment. The windowed VGRF signal is utilized for Parkinson’s disease binary classification in a number of deep learning backbone models through iterative subject-wise data segmentation. After that, we derive a reproducible walking biomarker through stability-based explainable artificial intelligence analysis and apply it to the prediction of Hoehn and Yahr severity through statistical verification.

Figure 1 shows the entire flow of the two-stage explainable artificial intelligence framework of this study based on the vertical ground reaction (VGRF) signal. This framework is designed to minimize information leakage through subject-wise data segmentation, integrate Parkinson’s disease diagnosis and severity prediction, and derive reproducible walking biomarkers.

The raw VGRF signals obtained from the PhysioNet (accessed on 10 October 2025) walking database are divided into fixed-length windows, and in each window, the mean and standard deviation are calculated for 16 plantar pressure sensors and left and right foot force signals to generate tokenized input representations. The data is then composed of training, verification, and test datasets through iterative subject-wise segmentation to reliably evaluate the generalization performance of the model [19].

In the first stage (Stage 1), Parkinson’s disease binary classification is performed using multiple deep learning backbone models such as TCN, BiGRU-Attn, and FCNN-Transformer, and performance is evaluated with indicators such as ROC-AUC, accuracy, sensitivity, and specificity. Subsequently, Integrated Gradients-based stability explainable artificial intelligence analysis is applied to consistently select important features across iterative segmentation and model structure as consensus explanatory features. These features are further verified through nonparametric statistical tests, effect size analysis, and FDR correction.

Finally, in the second stage (Stage 2), the XGBoost regression model is applied to subjects classified as Parkinson’s disease to predict the Hoehn & Yahr severity to evaluate whether the walking signal can simultaneously reflect the presence and progression of the disease.

### 2.1. Datasets and Preprocessing

In this study, we used gait data provided from the “Gait in Parkinson’s Disease Database” published on PhysioNet (accessed on 11 October 2025).

The dataset includes VGRF signals measured while walking for Parkinson’s disease patients and normal people.

This dataset is public data that has already been de-identified in the stage prior to being provided to the researcher and does not contain any information that may identify the individual. Therefore, this study corresponds to a secondary analysis study using de-identified public data, and no further deliberation by the Institutional Review Board (IRB) was required [5].

The raw VGRF signal was divided into fixed-length window units to effectively reflect the local temporal characteristics of walking and increase the number of learning samples. Specifically, the window length was 100 time steps, and an overlap ratio of 50% was applied to prevent loss of continuous walking characteristics. In addition, the signal was normalized using Z-score normalization to correct scale differences between sensors before model input. It is designed to prevent subject-level data leakage between learning, verification, and test datasets by maintaining the subject identifier in each window.

For each window, statistical summary features were calculated for 16 plantar pressure sensor signals and total left and right force signals. Specifically, the mean and standard deviation were calculated for each channel, and as a result, an input representation consisting of a total of 18 tokens (16 sensors + left and right foot forces) and two-dimensional features per token were generated. This tokenized input structure is designed to be suitable for sequence-based deep learning models while simultaneously preserving the magnitude and variability information of the walking force.

All data preprocessing and feature generation processes were performed in the Python 3.9 environment, and standard scientific calculation libraries such as NumPy and Pandas were used.

### 2.2. Subject-Wise Iterative Data Segmentation

In order to reliably evaluate the generalization performance of the model and prevent subject-level data leakage, the subject-wise iterative data segmentation strategy was applied in this study. If subject identifiers exist, group-based segmentation was performed so that all window data of the same subject was included only in one of the training, verification, and test datasets.

In each repeated experiment, the total data was divided into training data (70%), verification data (15%), and test data (15%) for each subject, and a total of five repeated evaluations were performed using different random seeds. All performance indicators were summarized as mean and standard deviation based on the results of the repeated experiments, and the 95% confidence interval was calculated using an empirical method based on the performance distribution obtained from repeated subject-wise evaluations.

Data segmentation and performance evaluation procedures were implemented using the scikit-learn 1.3 library.

### 2.3. Deep Learning Model for Binary Classification of Parkinson’s Disease

To distinguish Parkinson’s disease patients from normal people, three deep learning models were compared and evaluated: temporal convolutional network (TCN), BiGRU-Attn with attention mechanism, and FCNN-Transformer.

All models were implemented using the PyTorch 2.0 framework and learned using the AdamW optimizer. To alleviate the class imbalance problem, a weighted binary cross-entropy loss function was applied, and an early termination technique was used based on the AUC of the verification data.

### 2.4. A Two-Stage Framework for Severity Prediction

After the binary classification stage, Hoehn & Yahr (H&Y) severity was predicted through a regression-based model only for subjects classified as Parkinson’s disease. An XGBoost regression model based on gradient boosting was used to reflect the continuity of disease progression. The severity regression model was also configured to maintain subject-wise division so that the same subjects between learning and test data did not overlap.

Severity prediction performance was assessed using mean absolute error (MAE), root mean square error, and ranking-based correlation indicators to assess whether or not to preserve step order. Severity regression model was implemented using the XGBoost 1.7 library.

### 2.5. Stability-Based Explained Artificial Intelligence Analysis

For explanatory artificial intelligence analysis, the Integrated Gradients technique was applied to the learned deep learning model to calculate the contribution to each input token and feature dimension. Explanatory analysis was performed throughout iterative subject-wise segmentation and different model structures. The stability of feature importance was defined based on the frequency at which the same features were consistently selected throughout different model structures and iterative segmentation. In this study, the characteristics showing the contribution within the top 20% in each repetitive experiment and model were considered important. The percentage of all five experiments (number of repetitive divisions × number of models) in which the feature was selected as an important feature was quantified as a stability score, and a selection threshold was applied to select only the feature with a score of 20% or more as the consensus features. Explainability analysis was implemented using the Captum 0.6 library.

### 2.6. Statistical Validation

Statistical analysis was performed using the SciPy 1.10 library. In order to evaluate the difference in classification performance between models, a paired Wilcoxon signed-rank test was performed on the AUC value calculated in repetitive subject-wise segmentation. Differences between groups were evaluated by the Mann–Whitney U test, and the effect size was calculated using Cliff’s delta. To correct multiple comparison problems, FDR correction of the Benjamini–Hochberg method was applied.

In addition, a post-test power analysis was performed to verify the statistical validity of the two-stage severity regression analysis. The explanatory power of the regression model was evaluated based on the coefficient of determination (*R*^2^), and the effect size (*f*^2^) was calculated using the following equation.

(1) $$f^{2}=\frac{R^{2}}{1-R^{2}}$$

The significance level (α) was set to 0.05. The statistical power (1 − β) of regression analysis was calculated based on the calculated effect size, number of samples, and number of predictors to evaluate whether the dataset size was sufficient to support the analysis results.

All performance evaluations were performed under the strict subject-wise validation strategy to prevent data leakage.

## 3. Results

### 3.1. Technical Statistics Analysis of Datasets

A total of 166 participants were included in this study, of which 93 patients with Parkinson’s disease and 73 patients in a normal control group were included. As shown in Table 1, the average age of all subjects was 65.3 ± 8.1 years, and the gender distribution was relatively balanced with 88 males (53.0%) and 78 females (47.0%). The average age of the Parkinson’s disease patient group was 66.3 ± 8.2 years, and the average age of the normal control group was 64.1 ± 7.8 years; the age difference between the two groups was not statistically significant (*p* > 0.05). This suggests that the confounding effect according to age was minimized in the subsequent analysis.

The average Hoehn & Yahr level of the Parkinson’s disease patient group was 2.3 ± 0.6, and most of the subjects were in the mild to moderate level. In addition, the average Unified Parkinson’s Disease Rating Scale (UPDRS) score was 23.6 ± 9.4, indicating that this dataset was clinically composed mainly of Parkinson’s disease patients in the relatively early and intermediate stages.

As a result of evaluating functional mobility and walking ability, the average Timed Up and Go (TUG) test time of the Parkinson’s disease patient group was 13.2 ± 5.8 s, and the average walking speed (Speed_01) was measured as 0.86 ± 0.21 m/s. These walking indicators showed a significantly decreased pattern compared with the normal control group (*p* < 0.001) and quantitatively reflected the walking decline and motor dysfunction observed in Parkinson’s disease patients.

All gait data were collected through a VGRF sensor attached to both soles of the foot, and the signal was recorded at a sampling frequency of 100 (100 Hz) per second. Such a high-resolution time signal can accurately capture the distribution and variability of forces within each walking cycle, providing sufficient temporal information for deep learning-based analysis and explainable AI analysis performed thereafter. This dataset was used to comprehensively analyze the walking characteristics of Parkinson’s disease patients from a clinical and biomechanical perspective through comparison with normal controls.

### 3.2. Subject-Wise Parkinson’s Disease Binary Classification Performance

Parkinson’s disease binary classification performance was evaluated based on the subject-wise iterative data segmentation strategy, and data from the same subject were strictly controlled so that they were not included in the learning and evaluation stages at the same time. This evaluation strategy is intended to prevent performance overestimation due to overlapping subjects between training and verification data and to more reliably reflect generalization performance in real clinical settings.

As shown in Table 2, all three models recorded an average AUC of 0.93 or higher, confirming that Parkinson’s disease patients and normal controls can be effectively distinguished. Accuracy, sensitivity, and specificity also showed an overall balanced level, and stable classification performance was observed throughout the repeated experiments. In particular, the FCNN-Transformer model showed the highest discrimination performance with an average AUC of 0.940, and the BiGRU-Attn model showed relatively small standard deviation, showing excellent performance stability throughout repetitive segmentation.

In order to evaluate the statistical reliability of the model performance more quantitatively, a 95% confidence interval (95% CI) was calculated for the AUC value obtained in the repeated experiments. As a result, the AUC of the FCNN-Transformer model was 0.940 (95% CI: 0.912–0.968), the BiGRU-Attn model was 0.934 (95% CI: 0.917–0.951), and the TCN model was 0.934 (95% CI: 0.881–0.987), confirming that all three models stably maintained a clinically meaningful level of discrimination performance.

In addition, to evaluate whether the performance difference between the FCNN-Transformer model and other models was accidental, a paired Wilcoxon signed-rank test was performed on the AUC value obtained from the same subject-wise segmentation. As a result, the FCNN-Transformer model showed a statistically significantly higher AUC compared with the TCN model and the BiGRU-Attn model (*p* < 0.05, respectively). This means that the performance excellence of the FCNN-Transformer model was consistently maintained throughout the iterative segmentation.

On the other hand, in the BiGRU-Attn model, the average AUC was slightly lower than that of FCNN-Transformer, but the balance between sensitivity and specificity was the best and the performance volatility was the smallest, so it was evaluated as a competitive model in terms of clinical stability. On the other hand, the TCN model showed relatively large performance volatility according to repeated divisions, and some divisions showed high sensitivity or specificity, but performance degradation was confirmed in other divisions. These results suggest that subject composition differences in windowed VGRF data can have a real impact on model performance, supporting the need for subject-wise iterative evaluation strategies.

It should be noted that, as a result of the confusion matrix analysis, no clinically fatal misclassification was observed in all models that classified the normal control group as Parkinson’s disease or patients with Parkinson’s disease as normal. This suggests that the windowed VGRF-based input representation used in this study stably captures the typical walking characteristics of Parkinson’s disease.

Taken together, the approach of this study, which combines a windowed VGRF-based input representation and subject-wise iterative evaluation strategy, simultaneously provides high classification performance, statistically verified differences between models, and good generalizability in Parkinson’s disease binary classification.

### 3.3. Results of Stability-Based Explainable Artificial Intelligence (XAI) Analysis

Stability-based explainable AI analysis was performed to identify consistently important walking features across iterative subject-wise data segmentation and different deep learning backbone models. Integrated Gradients (IGs)-based contribution analysis was applied to each of the TCN, BiGRU-Attn, and FCNN-Transformer models for this purpose, and the feature importance calculated from the test data was aggregated between repetitive experiments and between models.

**Figure 2:**
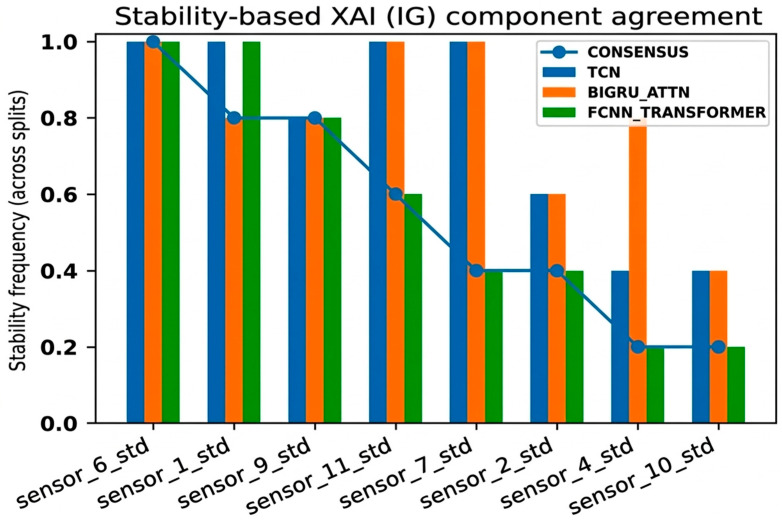
Stability-based XAI (Integrated Gradients) component agreement across models and subject-wise splits.

Figure 2 is the result of the stability frequency, showing how consistently the importance of each walking feature was selected throughout the repetitive segmentation and model structure. Here, the stability frequency refers to the ratio at which the same feature was selected as an important feature throughout the different model structures compared with the repetitive subject-wise segmentation. As a result, features showing a high stability frequency in common across the three models, rather than features limited to a single model or a specific segmentation, were identified. In particular, it was clearly observed that variability-based features such as standard deviation (std) tended to be concentrated above the mean-based features, suggesting that the temporal stability of Parkinson’s disease patients’ walking is lower than that of the normal control group.

After selecting the upper contributing features based on these stability-based XAI results, statistical verification was performed to confirm whether the features showed differences between groups even at the actual data level. Differences between groups were evaluated by the Mann–Whitney U test, and the effect size was calculated as Cliff’s delta. In addition, false discovery rate (FDR) correction was applied to correct type 1 errors due to multiple comparisons.

Table 3 presents a quantitative summary of the final candidate biomarkers that have passed statistical verification among gait features selected through stability-based XAI analysis. For each feature, the median and quartile range (IQR) of the normal control group and Parkinson’s disease patient group, the FDR-corrected *p*-value, and the effect size (Cliff’s delta) are presented together to clearly show whether model-based importance results lead to actual data distribution differences.

**Figure 3:**
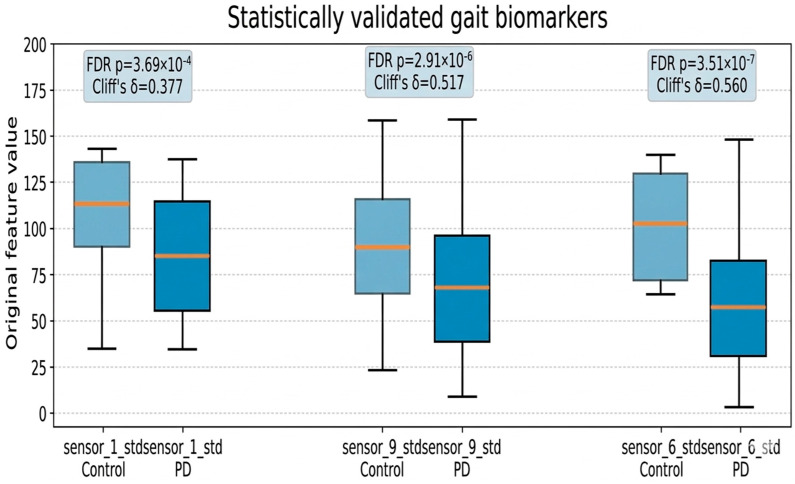
Statistically validated gait biomarkers distinguishing Parkinson’s disease from controls.

Figure 3 is the result of visualizing the original data distribution of the normal control group and the Parkinson’s disease patient group with a boxplot for the statistically significant features presented in Table 3. As a result, vertical ground reaction variability-based features such as sensor_1_std, sensor_9_std, and sensor_6_std showed a statistically significant difference between groups even after FDR correction (*p* < 0.001) and showed a moderate or higher effect size.

In particular, in all significance features, the Parkinson’s disease patient group showed a tendency to decrease the standard deviation value compared with the normal control group, which can be interpreted as a result reflecting the rigidity of the ground reaction force control and the reduction of rhythmic diversity when walking. Taken together, the stability-based XAI analysis proposed in this study demonstrated that it is possible to identify reproducibly derived gait features across repetitive segmentation and multi-model structures, and these features have the potential as a candidate for a walking biomarker capable of statistically verified clinical interpretation.

### 3.4. Hoehn & Yahr Severity Regression Prediction Results

To evaluate whether the progression of the disease can be quantitatively predicted beyond the presence or absence of Parkinson’s disease, in this study, regression analysis of Hoehn & Yahr (H&Y) severity was performed only for subjects who were identified as Parkinson’s disease in the binary classification stage. In this step, we trained the XGBoost-based regression model using the windowed VGRF-based gait feature as input. In particular, even at this stage, strict subject-wise segmentation conditions were maintained to prevent the same subject from being included between the training data and the test data in the same way as binary classification. This ensured that the high predictive performance was not due to the overfitting of the subject of the model.

**Figure 4:**
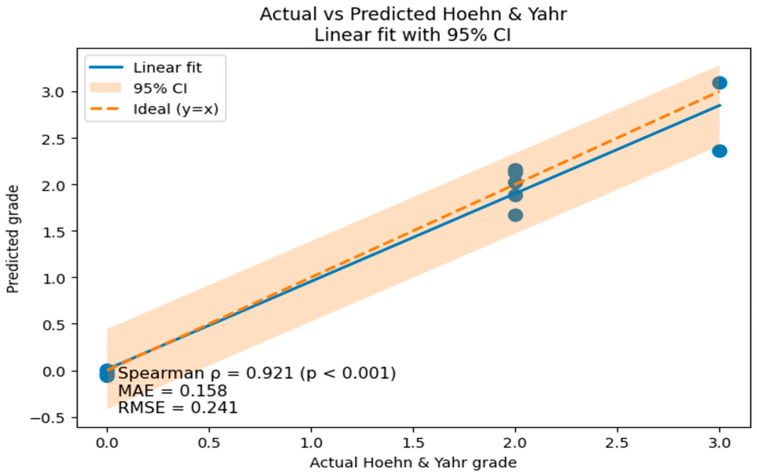
Actual versus predicted Hoehn & Yahr stages with linear regression and 95% confidence interval.

Figure 4 shows the results of visualizing the relationship between the actual H&Y grade and the continuous severity value predicted by the regression model. Each blue point represents an individual subject’s prediction, indicating the relationship between the actual clinical grade and the corresponding model-predicted severity value. As a result of a scatterplot analysis, the predicted value showed a strong positive correlation with the actual clinical grade, and the linear regression line was distributed close to the ideal prediction line (y = x). In particular, the Spearman correlation coefficient was ρ = 0.921 (*p* <0.001), confirming that this regression model preserves the sequence relationship of H&Y severity at a very high level.

As a result of quantitatively evaluating the prediction accuracy, the mean absolute error (MAE) was 0.158 and the root mean squared error (RMSE) was 0.241, confirming that the severity was generally predicted within the error range of about 0.2 steps compared with the actual clinical stage. In addition, the coefficient of determination (*R*^2^) was 0.953, showing very high explanatory power that the regression model explained 95.3% of the actual Hoehn & Yahr severity variability. The 95% confidence interval (95% CI) displayed around the regression line also remained relatively narrow in the entire section, suggesting that the uncertainty of model prediction is limited.

Table 4 presents representative examples comparing the actual H&Y grade and the severity value predicted by the regression model. These cases are presented to illustrate the prediction characteristics of the model rather than to summarize the entire test set. In cases falling under H&Y stage 0, the predicted value ranged from −0.06 to 0.01, which was very close to the actual value, and in most cases, the prediction error was limited to within 0.5 in H&Y stages 2 and 3. A relatively large error was observed in some cases, but this also occurred within the range of adjacent stages and corresponds to a clinically interpretable level.

It should be noted that by predicting the Hoehn & Yahr grade, for a discrete stage scale, as a continuous value, a slight difference in severity could be quantitatively expressed even within the same clinical stage. This means that the existing categorical severity classification method can complement the continuous characteristics of disease progression that are difficult to capture.

Taken together, the combination of the windowed VGRF-based gait feature and the XGBoost regression model proposed in this study presents the possibility of continuous estimation of disease severity as well as the presence of Parkinson’s disease. This step-by-step analysis strategy of binary classification–severe regression is expected to be used as an integrated clinical decision-making aid to simultaneously support the diagnosis and progression monitoring of Parkinson’s disease in the future.

## 4. Discussion

### 4.1. Clinical Implications of Binary Classification Performance Based on Topic-Specific Iterative Validation

In this study, a subject-wise iterative data segmentation strategy was applied to evaluate the Parkinson’s disease binary classification performance [23]. Specifically, a training/verification/evaluation set was constructed based on the subject ID by performing segmentation according to the subject exclusion principle described in reference [23], and all samples (including all walking sections/window and session) extracted from one subject were assigned to only one set. This thoroughly excluded the possibility of data leakage in which data from the same subject was included in the learning and evaluation stages simultaneously. This is an approach to fundamentally block the problem of performance overestimation due to subject-level data leakage, which has been frequently pointed out in existing gait-based Parkinson’s disease classification studies. Under this evaluation strategy, all three deep learning backbone models (TCN, BiGRU-Attn, and FCNN-Transformer) showed high classification performance above the average AUC of 0.93, suggesting that the windowed VGRF-based walking signal contains enough information to effectively distinguish the presence or absence of Parkinson’s disease.

In particular, the FCNN-Transformer model recorded the highest average AUC in the overall repetitive segmentation and showed a statistically significant performance advantage over other models in the paired Wilcoxon signed-rank test results. This means that the self-attention-based structure was effective in simultaneously learning the long- and short-term dependence between time sections. On the other hand, the BiGRU-Attn model has a slightly lower average performance but has the smallest variance and can be evaluated as a model with high clinical applicability in terms of performance stability. These results suggest that the importance of stability and reproducibility required in a clinical environment should be considered together beyond simply presenting the “highest performance model”.

### 4.2. Strengths of Windowed VGRF-Based Input Representation

The windowed VGRF input representation used in this study is designed to effectively reflect the variability between repetitive walking cycles while maintaining the temporal structure of the primitive walking signal. Existing gait analysis studies often relied on single walking cycle averages or summary statistics, but this approach has a limitation in that it does not sufficiently capture the microscopic temporal instability in Parkinson’s disease patients [19].

The classification and explainability analysis results of this study show that the windowed VGRF expression effectively reflects characteristics such as decreased rhythm, stiffness of force control, and decreased temporal variability in Parkinson’s disease patients’ walking. In particular, the fact that differences in variability patterns within the walking cycle acted as an important clue to the model classification, even if they showed the same walking speed or average force level, provides important implications to complement the limitations of simple mean-based analysis.

### 4.3. Significance of Stability-Based XAI Analysis and Reproducibility of Biomarker Candidates

In explainable AI analysis, this study does not depend on the importance of features derived from a single model or a single data segmentation but rather selects features consistently derived from repetitive subject-wise segmentation and different deep learning backbones based on stability [24]. This approach is a key strategy for securing the reproducibility and reliability of XAI results, which alleviates the limitation of “model-dependent explanatory results” that has been pointed out in previous studies.

As a result, variability-based VGRF features such as standard deviation (std) showed higher stability in common than mean-based features. This is consistent with the biomechanical properties that Parkinson’s disease patients’ walking shows a more regular and rigid pattern compared with the normal control group, while relatively diverse force distributions and rhythmic variability are maintained in normal walking. In addition, these features showed statistically significant differences between groups even after the Mann–Whitney U test and FDR correction and showed moderate or higher effect size, supporting that they are biomarker candidates based on differences in actual data distribution, not just model outputs.

### 4.4. Possible Clinical Expansion of Hoehn & Yahr Severity Regression Prediction

An important expansion point of this study is the attempt to continuously predict disease severity beyond the presence or absence of Parkinson’s disease. The Hoehn & Yahr scale is a clinically widely used severity indicator, but it has a limitation in that it does not sufficiently reflect minute differences between patients in the same stage because it is composed of discrete stages.

As a result of XGBoost-based regression analysis, the predicted severity value showed a high sequence correlation (Spearman ρ = 0.921) with the actual H&Y stage, and a high explanatory power of *R*^2^ 0.953 was recorded with a low error of 0.158 for MAE and 0.241 for RMSE. This means that walking-based features alone can significantly explain the variability in clinical severity. This regression model presents the potential as a tool for continuous severity estimation that complements the existing staged evaluation, given that it has secured ranking preservation ability and numerical accuracy at the same time.

### 4.5. Binary Classification Clinical Implications of Severe Regression Integration Strategy

The analysis strategy of this study, which combines binary classification and severity regression in stages, is significant in that it can integrally support the diagnosis and progression monitoring of Parkinson’s disease within one framework [19]. In the actual clinical environment, not only the presence or absence of the disease, but also the extent to which the disease has progressed has an important influence on treatment strategy decision and prognosis evaluation.

The results of this study show that a windowed VGRF-based gait analysis can meet these two needs simultaneously, suggesting the possibility of expanding to digital biomarker-based remote monitoring or long-term follow-up studies in the future. In particular, continuous severity prediction can be used for tracking changes over time of the same patient, so it has meaning as an objective indicator that complements the qualitative evaluation of existing clinical scales.

### 4.6. Limitations of Research and Future Research Directions

There are several limitations to this study. First, the dataset of this study is composed mainly of mild and moderate patients in stages 1 to 3, so there is a class imbalance in the sample. Due to these distributional characteristics, the high regression prediction performance (*R*^2^ = 0.953) of this model can be the result of reflecting the pattern of patients with early and middle Parkinson’s disease, and it has the potential to underestimate the prediction performance or cause errors in patients with severe high risk of H&Y level 4 or higher. Second, since the proposed model is optimized for the VGRF signal based on a specific plantar pressure sensor system, additional cross-validation is required to determine whether the framework can be generalized equally to gait data collected in other environments, such as smart insole or smartphone, or to other neurological disease groups. Second, since this study is based on cross-sectional data, it was not possible to directly analyze the long-term disease progression of the same patient.

In future studies, it is necessary to further verify the generalizability and temporal sensitivity of the proposed framework by utilizing large datasets and longitudinal data including more diverse severity distributions. In addition, by integrating clinical variables, drug use information, or wearable sensor-based multi-modal data, it could be extended to more precise severity estimation and prognosis prediction.

Taken together, this study presented an integrated approach that simultaneously secured reliability, reproducibility, and clinical interpretability in the Parkinson’s disease gait analysis study by combining subject-wise iterative verification, stability-based explainable artificial intelligence, and severity regression analysis. These results suggest that walking-based digital biomarkers can substantially contribute to the diagnosis and evaluation of progression of Parkinson’s disease, supporting the possibility of future use as a clinical decision-making assistance system.

## 5. Conclusions

In this study, we proposed an explainable AI-based integrated analysis framework that can simultaneously evaluate the presence or absence of Parkinson’s disease and disease severity using VGRF-based gait signals. The proposed approach applies to a subject-wise iterative data segmentation strategy to prevent performance overestimation due to data duplication of the same subject and reliably evaluate the generalizability in real clinical settings.

In the Parkinson’s disease binary classification stage, the comparison and evaluation of the three deep learning backbone models of the temporal convolutional network (TCN), BiGRU-Attn, and FCNN-Transformer confirmed the excellent classification performance above the average AUC of 0.93 in all models. In particular, the FCNN-Transformer model showed statistically significantly high classification performance, and the BiGRU-Attn model showed the most stable performance throughout repetitive segmentation, suggesting clinical applicability. These results show that the windowed VGRF-based input representation can effectively reflect Parkinson’s disease walking characteristics.

Through Integrated Gradients-based stability analysis, we identified gait features that can be reproducibly derived from repetitive segmentation and across different model structures in explainable AI analysis. As a result, standard deviation-based features that reflect the variability of vertical ground reaction force rather than average-based features were found to be key contributing factors, and these features showed significant differences between groups even at the actual data level through statistical verification. This suggests that model-based importance results can be linked to clinically interpretable walking biomarker candidates, not just computational products [22].

In addition, this study suggested the possibility of supplementing the existing discrete stage evaluation by continuously predicting the severity of Hoehn & Yahr by applying the XGBoost regression model to subjects classified as Parkinson’s disease. As a result of the regression analysis, the predicted value showed a high sequence correlation with the actual clinical severity and achieved low predictive error and high explanatory power. This means that walking-based features alone can explain the variability in disease severity to a large extent.

Taken together, this study simultaneously secured the reliability, reproducibility, and clinical interpretability of Parkinson’s disease gait analysis through an integrated framework that combines subject-wise verification, stability-based explainable artificial intelligence, and severity regression analysis. The proposed approach has the potential to be used as an objective digital biomarker-based decision-making aid to support the diagnosis and monitoring of Parkinson’s disease and is expected to further expand its clinical applicability through large-scale and longitudinal studies in the future.

The proposed approach suggests its potential as an objective decision-making aid supporting the diagnosis and monitoring of progression of Parkinson’s disease. However, a somewhat cautious approach is required for the results of this study to be applied independently and directly to the real-world clinical setting. In the future, follow-up studies that demonstrate the clinical reliability of this digital biomarker through external validation in different hospital cohorts and longitudinal studies that track changes in the patient’s condition for a long time are essential.

## Figures and Tables

**Table 1:** Demographic characteristics and clinical severity of Parkinson’s disease patients and normal controls.

Group	N	Mean Age (SD)	Male (N)	Female (N)	Hoehn & Yahr
Control (CO)	73	64.1 ± 7.8	38	35	-
Parkinson’s Disease (PD)	93	66.3 ± 8.2	50	43	2.3 ± 0.6

**Table 2:** Parkinson’s disease title classification performance.

Model	AUC (Mean ± SD)	AUC (95% CI)	ACC (Mean ± SD)	Sensitivity (Mean ± SD)	Specificity (Mean ± SD)
TCN	0.934 ± 0.061	0.881–0.987	0.836 ± 0.068	0.825 ± 0.116	0.864 ± 0.103
BiGRU-Attn	0.934 ± 0.025	0.917–0.951	0.847 ± 0.037	0.843 ± 0.078	0.866 ± 0.095
FCNN-Transformer	0.940 ± 0.047	0.912–0.968	0.862 ± 0.045	0.854 ± 0.079	0.879 ± 0.064

**Table 3:** Stability-based XAI-derived gait features with statistical validation.

Feature	Control Median (IQR)	PD Median (IQR)	FDR-Adjusted *p*-Value	Cliff’s Delta (PD-CO)
sensor_1_std	113.93 (52.87)	75.06 (60.32)	<0.001	−0.446
sensor_9_std	110.43 (46.43)	78.87 (63.93)	<0.001	−0.436
sensor_6_std	80.36 (34.44)	75.03 (31.45)	<0.001	−0.153

**Table 4:** Comparison of actual and predicted stages.

Feature	Actual H&Y	Predicted Grade	Absolute Difference
1	0	−0.055	0.055
2	3	3.093	0.093
3	2	1.887	0.113
4	0	−0.037	0.037
5	2	2.024	0.024
6	2	2.158	0.158
7	2	2.130	0.130
8	3	2.365	0.635
9	2	1.669	0.331
10	0	0.006	0.006

## Data Availability

All data supporting the findings of this study are publicly available in the GaitPDB database on PhysioNet (https://physionet.org/content/gaitpdb/1.0.0/) (accessed on 1 October 2025). No new data were generated during this study.
